# Nutritional value, chemical composition and antioxidant activity of three *Tuber* species from China

**DOI:** 10.1186/s13568-017-0431-0

**Published:** 2017-06-26

**Authors:** Xiangyuan Yan, Yanwei Wang, Xiaoyu Sang, Li Fan

**Affiliations:** College of Life Science, Capital Normal University, Beijing, 100048 People’s Republic of China

**Keywords:** Truffle, Nutrients, Bioactive compounds, DPPH, Reduce power

## Abstract

Nutritional value, chemical composition and antioxidant activity of the traditional edible truffles *Tuber latisporum*, *T. subglobosum* and *T. pseudohimalayense*, from China were evaluated. Powder formulations of the three truffles revealed the presence of essential nutrients, such as proteins, carbohydrates and unsaturated fatty acids, and *T. latisporum* presented the highest contents of total sugar (50.10 g/100 g) and monounsaturated fatty acids (265.19 mg/100 g dw); *T. pseudohimalayense* showed the highest content of polyunsaturated fatty acids (367.98 mg/100 g dw). They all presented a low fat content but high contents of proteins and unsaturated fatty acid, which is beneficial to human health. The methanol extract from *T. pseudohimalayense* showed a high radicals scavenging activity and the highest content of total phenols (735.01 mg/100 g dw); *T. subglobosum* presented the highest content of flavonoids (1355.43 mg/100 g dw). All these extracts could be used as potential antioxidant sources to prevent diseases related to oxidative damage.

## Introduction

Mushrooms are a popular food in the world and they have become very attractive due to their low calories, fats, and essential fatty acids (FA), and rich vegetable proteins, vitamins and minerals (Wang and Marcone 2011; Hamza et al. 2016). Truffles, a group of hypogenous mushroom belonging to the Ascomycota, were particularly appreciated for their unique aroma, great economic value and potential health benefits (Hall et al. 2003; Beara et al. 2014). In fact, truffles were rich in unsaturated fatty acids (UFA), some kind of free sugars that are good for human health, and therapeutic compounds with anti-inflammatory, antioxidant, antimicrobial, anti-mutagenic and anti-carcinogenic properties (Bokhary and Sarwat 1993; Carneiro et al. 2013; Culleré et al. 2010; Dundar et al. 2012; Gao et al. 2001a, b; Murcia et al. 2002; Saltarelli et al. 2008; Sawaya et al. 1985; Stojković et al. 2013).

A radical is any molecule or atom with one or more unpaired electrons, normally generated in many metabolic pathways in the human body. Some of them can exist in a free form and subsequently oxidize biological molecules such as lipids, DNA, carbohydrates and proteins, resulting in the damage to these molecules and the dysfuction of related tissue components (Kehrer 1993; Dubost et al. 2007). These free radicals have come to occupy a central role in a wide variety of diseases, such as atherosclerosis, cancer, rheumatoid arthritis and degenerative processes associated with aging (Lee et al. 2004). Some recent reports had shown the truffles, have the ability to scavenge free radicals because they contain various polyphenolic/phenolics, flavonoids or sterols compounds (Beara et al. 2014; Tang et al. 2012; Stojković et al. 2013; Villares et al. 2012), which are recognized as an antioxidant due to their ability to scavenge free radicals by acting as reducing agents, hydrogen donating antioxidants and singlet oxygen quenchers (Barros et al. 2007).

The three truffle species in this study, *Tuber latisporum*, *T. subglobosum* and *T. pseudohimalayense*, are also traditional edible mushrooms with a great production in China. Our aim was to increase the knowledge about their chemical composition and nutritional properties, with regard to the contents of energy, carbohydrate, protein, fat, ash, free sugar and fatty acid; to investigate the antioxidant activity of these truffles, including scavenging ability for DPPH and hydroxyl radicals, reduce power, chelating effect on ferrous ion. This is the first report on the chemical constituents and antioxidant activities of the three Chinese truffles. Gas chromatography–mass spectrometer (GC–MS) and high-performance liquid chromatography (HPLC) techniques were applied to evaluate composition analysis, the antioxidant potential was determined using various assays.

## Materials and methods

### Truffle samples

Fresh ascocarp samples of *T. latisporum*, *T. subglobosum* and *T. pseudohimalayense* were collected from local markets in Yunnan province of China and were authenticated by Dr. Li Fan (College of Life Science, Capital Normal University, PR China). All the truffles were identically selected in terms of shape, size, colour, and ripening stage. After collection and taxonomic identification, fresh truffles were selected and washed to remove contaminants, then freeze-dried (Wizard 2.0, VirTis, USA) at −45 °C. The lyophilized truffle samples were ground into fine powder (40 mesh) with a Mixer Mill (Retsch, Haan, Germany). The lyophilized truffle samples were stored in the dark at 4 °C until analysis.

### Standards and reagents

Acetonitrile 99.9%, *n*-hexane 95% and methanol 99.9% were purchased from Fisher Scientific (Shanghai, China). The fatty acids methyl ester (FAME) reference standard mixture 37 (standard 47885-U) was purchased from Sigma (St. Louis, MO, USA). Sugars standards [glucose, sorbitol and d(+)-trehalose and myo-inositol], phenolic standards (gallic, 4-hydroxybenzoic, 3,4-dihydroxybenzaldehyde, homogentisic, *p*-coumaric and *o*-coumaric acids) were purchased from Sigma (St. Louis, MO, USA) or Sigma-Aldrich Chem (shanghai, China). 1,1-Diphenyl-2-picryl-hydrazyl (DPPH), BHT, 3-(2-pyridyl)-5,6-bis (4-phenyl-sulfonic acid)-1,2,4-triazine (ferrozine), rutin and ascorbic acid were purchased from Sigma Chemical Co. BSTFA + TMCS (99:1) was purchased from Supelco. Folin–Ciocalteu’s phenol reagent was purchased from Beijing Solarbio reagent company (Beijing, China). Ferrous chloride, sodium salicylate, ferrous sulphate, hydrogen peroxide, potassium ferricyanide, trichloroacetic acid, disodium phosphate, sodium dihydrogen phosphate, ferric chloride, sodium carbonate, sodium hydroxide, aluminium nitrate, sodium nitrite, petroleum ether (60–90 °C), ethanol and methanol were all purchased from Beijing chemical plant (Beijing, China). All chemicals were of analytical grade. Water was treated in a Milli-Q water purification system (TGI Pure Water Systems, USA).

### Analysis of chemical composition

#### Nutritional value

The lyophilized truffle samples were analyzed for proteins, fat, carbohydrates and ash, using the AOAC (1995) procedures. The crude protein content (N × 4.38) of the samples was estimated by the macro-Kjeldahl method; the crude fat was determined by extracting a known weight of powdered sample with petroleum ether, using a Soxhlet apparatus; the ash content was determined by incineration at 600 ± 15 °C. Total carbohydrates were calculated by difference. Energy was calculated according to the following equation: energy (kcal) = 4 × (g proteins + g carbohydrates) + 9 × (g fat).

#### Sugars

The lyophilized truffle samples (2 g) were spiked with the Internal Standard (IS, raffinose, 5 mg/mL), and were extracted with 120 mL of 80% aqueous ethanol at 80 °C for 1 h 30 min. The resulting suspension was filtered and concentrated using a rotary evaporator (Laborota 4003, Heidolph WB, Germany) and defatted three times with 20 mL of petroleum ether, successively. After concentration, the residues were dissolved in water to a final volume of 1000 mL.

Sugars were determined after a silanization procedure performed. 200 μL dilute solution was evaporated to dryness over N_2_ then 100 μL BSTFA + TMCS (99:1, v/v) and 20 μL pyridine were added, reacting at 70 °C for 2 h. After 580 μL dichloromethane added, the sample was recovered in a vial with Teflon cap, and before injection the sample was filtered with 0.2 μm Nylon filter from Millipore.

The analysis was performed using GC–MS (Thermo Finnigan trace DSQ II) equipped with a fused silica HP-5MS capillary column (30 m × 0.25 mm ID × 0.25 μm df). 1 μL of sample was injected in a split/splitless injector set at 250 °C. The GC was setup with helium as a carrier gas at a constant flow of 1.0 mL/min. The column temperature program was: 50 °C (2 min); to 120 °C (5 min) at 15 °C/min; to 290 °C (10 min) at 5 °C/min. The mass spectrometer was used in electron impact mode, with the electron energy at 70 eV, ion source temperature at 290 °C, transfer line temperature was 290 °C, scan range m/z: 50–550 amu, data acquisition scan mode for full scan.

Sugars’ identification was made by comparing the relative retention times of sample peaks with standards. Quantification was based on the MS signal response of each standard, using the IS method and by using calibration curves obtained from commercial standards of each compound. The results were expressed in g per 100 g of dry weight.

#### Fatty acids

Fatty acids were determined after a transesterification procedure performed with the oil obtained by soxhlet extraction (extraction ratio: *T. latisporum* 2.40%; *T. subglobosum* 2.23%; *T. pseudohimalayense* 2.55%): fatty acids (2 mg) were methylated with 6 mL of 0.6 mol/L NaOH methanol solution: *n*-hexane 2:1 (v:v) with vortexing for 3 min. After 20 min in a bath at 30 °C, 3 mL of deionized water was added, to obtain phase separation; the upper phase was recovered in a vial, and before injection the sample was filtered with 0.2 μm Nylon filter from Millipore. The analysis was performed using GC–MS equipped with a fused silica HP–5MS capillary column (30 m × 0.25 mm ID × 0.25 μm df). The column temperature program was: 45 °C (1 min); to 175 °C (2 min) at 10 °C/min; to 210 °C (5 min) at 1 °C/min; to 280 °C (1 min) at 5 °C/min. 1 μL of sample was injected.

### Analysis of chemical composition in bioactive compounds

#### General

The sample (15 g) was stirred with methanol (300 mL), sonicated for 30 min and incubated at 35 °C for 1 h. Then, the methanol extract (ME) was centrifuged at 5000*g* for 15 min at 35 °C. The residue was then re-extracted under the same conditions. The combined extracts were filtered through Whatman No. 1 filter paper; the filtrate was collected and evaporated using a rotary evaporator, weighed and re-dissolved in methanol at 50 mg/mL (stock solution), and stored in the dark at 4 °C for further use. Successive dilutions were made from the stock solution and submitted to different in vitro assays to evaluate the content of active compounds and antioxidant activity of the samples.

#### Total phenols

1 mL ME (3 mg/mL) was mixed with 1 mL of Folin–Ciocalteu’s phenol reagent; after 3 min, Na_2_CO_3_ (35%, 1 mL) was added, and then distilled water was added making the reaction system reach 10 mL. The reaction mixture was mixed thoroughly and allowed to stand for 90 min at room temperature in the dark. Absorbance of all the sample solutions against a blank was measured at 725 nm using the spectrophotometer (Lambda 35, Perkin Elmer Co. Ltd., USA). Total phenolic contents were expressed as mg gallic acid (Sigma) equivalents, GAE/100 g of extract. Calibration curve was constructed with different concentrations of Gallic acid (1–12 μg/mL) as the standard. The assays were carried out in triplicate; total phenolic contents were mean values ± standard deviations.

#### Total flavonoids

Total flavonoids of ME were measured by the method of Guo et al. (2011). A calibration curve was constructed with different concentrations of rutin (0.01–0.1 mg/mL) as the standard. The assays were carried out in triplicate; total flavonoid contents were expressed on an extract weight basis as mg/g rutin equivalents, rutin mg/100 g of extract.

#### VC

Ascorbic acid was determined according to the method of Klein and Perry (1982) with slight modification in the content of metaphosphoric acid in extraction. 100 mg ME was extracted with 10 mL of 1% metaphosphoric acid for 45 min at room temperature and filtered through Whatman No. 4 filter paper. The filtrate was mixed with 2,6-dichlorophenolindophenol (1v:9v) and the absorbance was measured at 515 nm against a blank within 30 min. A calibration curve was constructed with different concentrations of authentic l-ascorbic acid (0.020–0.12 mg/mL) as the standard. The assays were carried out in triplicate; the results were mean values ± standard deviations and expressed in g/100 g of dry weight.

#### β-Carotene and lycopene

β-Carotene and lycopene were determined by the following process (Barros et al. 2007). 100 mg ME was vigorously shaken with 10 mL of acetone–hexane mixture (4:6) for 1 min. The absorbance of the filtrate was measured at 453, 505 and 663 nm. Contents of β-carotene and lycopene were calculated according to the following equations: lycopene (mg/100 mL) = −0.0458A_663_ + 0.372A_505_ − 0.0806A_453_; β-carotene (mg/100 mL) = 0.216A_663_ − 0.304A_505_ + 0.452A_453_. The assays were carried out in triplicate; the results were mean values ± standard deviations and expressed in mg/100 g of dry weight.

#### Phenolic compounds

Phenolic compounds were analyzed using a Hewlett–Packard 1200 chromatograph (Agilent Technologies) with a quaternary pump and a diode array detector (DAD) coupled to an HP Chem Station (rev. A.05.04) data-processing station. The solvents used were: (A) 0.1% acetic acid in water, (B) acetonitrile. The elution gradient established was 5% B to 5% B over 5 min, 5–33% B over 15 min, 33–80% B over 10 min, 80–100% B over 5 min, isocratic 100% B for 5 min, and re-equilibration of the column, using a flow rate of 1 mL/min. Double online detection was carried out in the DAD using 280 and 370 nm as preferred wavelengths. The phenolic compounds were characterized according to their UV retention times, and comparison with authentic standards when available. For quantitative analysis, calibration curves were prepared from different standard compounds (r^2^ > 0.999). The assays were carried out in triplicate; the results were expressed in mg/100 g of dry weight.

### Evaluation of antioxidant activity

#### Ferrous ion-chelating assay

Ferrous ion-chelating activities of ME were evaluated by the following process (Guo et al. 2011). An aliquot of each sample (1 mL), with different concentrations, was mixed with FeCl_2_ (2.0 mM, 0.1 mL) and methanol (3.7 mL). The reaction was initiated by adding ferrozine (5.0 mM, 0.2 mL), and incubated at 35 °C for 20 min. The absorbance of the mixture was determined at 562 nm.

#### Ferricyanide/Prussian blue assay

Ferrous ion-chelating activities of ME were evaluated by the method of Guo et al. (2011). Solutions of the ME at different concentrations (0.2 mL) were mixed with sodium phosphate buffer (200 mmol/L, pH 6.6, 0.5 mL) and potassium ferricyanide (1% w/v, 0.5 mL). The mixture was incubated at 50 °C for 20 min, and trichloroacetic acid (10% w/v, 0.2 mL) was added, followed by centrifugation at 3000*g* for 10 min. The supernatant (0.5 mL) was mixed with deionized water (0.5 mL) and ferric chloride (0.1% w/v, 0.1 mL); then the absorbance was measured at 700 nm.

#### DPPH scavenging activity assay

Aliquots (0.1 mL) of various concentrations (0–30 mg/mL) of ME were mixed with 0.9 mL (25 μg/mL) of a MeOH solution of DPPH. The mixture was shaken and left in the dark for 30 min (Guo et al. 2011). The absorbance was measured with a spectrophotometer at 517 nm against a blank.

#### Hydroxyl radical-scavenging assay

Hydroxyl radical-scavenging activities of ME were determined according to the method described by Guo et al. (2011). Sodium salicylate (20 mM, 0.3 mL), FeSO_4_ (1.5 mM, 1.0 mL), various concentrations of sample solution (1.0 mL) and H_2_O_2_ (6.0 mM, 0.7 mL) were mixed immediately and left at 37 °C for 1 h. The absorbance of the mixture was recorded at 510 nm.

### Statistical analysis

All the assays were carried out in triplicate. The results are expressed as mean values ± SD (standard deviations). Statistical analyses were performed using a one-way analysis of variance ANOVA test and the significance of the difference between means was determined by Duncan’s multiple range test. Differences at p < 0.05 were considered statistically significant. This treatment was carried out using SPSS v. 16.0 program.

## Results

### Chemical composition in nutritional compounds

The results of the macronutrients, estimated energetic value and individual sugars of the studied lyophilized truffle samples are shown in Table 1. Carbohydrates were the most abundant macronutrients, followed by proteins; fat and ash contents were low. Their carbohydrate contents were close to each other, respectively 74.63% in *T. latisporum*, 78.68% in *T. subglobosum* and 74.40% in *T. pseudohimalayense*; the contents of protein in white truffle *T. latisporum* (14.64%) and red truffle *T. pseudohimalayense* (14.28%) were higher than that of pale yellow truffle *T. subglobosum* (10.96%); the fat contents in all three truffles can be as low as 2.23–2.55%.

**Table 1:** Macronutrients and free sugars composition in dried powder formulations of three *Tuber* species

	*T. latisporum*	*T. subglobosum*	*T. pseudohimalayense*
Ash^d^	8.33 ± 0.23^b^	8.13 ± 0.23^b^	8.77 ± 0.12^a^
Proteins^d^	14.64 ± 0.52^a^	10.96 ± 0.35^b^	14.28 ± 0.95^a^
Fat^d^	2.40 ± 0.03^b^	2.23 ± 0.04^c^	2.55 ± 0.07^a^
Carbohydrates^d^	74.63 ± 0.39^b^	78.68 ± 0.17^a^	74.40 ± 0.88^b^
Energy^e^	378.64 ± 0.79^a^	378.60 ± 0.74^a^	377.71 ± 0.70^a^
Glucose^d^	1.25 ± 0.18^a^	0.92 ± 0.02^b^	1.22 ± 0.05^a^
Trehalose^d^	13.84 ± 1.02^a^	4.41 ± 0.59^b^	4.22 ± 0.15^b^
Sorbitol^d^	33.82 ± 3.02^a^	22.65 ± 2.15^b^	17.55 ± 0.36^c^
Inositol^d^	1.18 ± 0.05^b^	2.00 ± 0.06^a^	0.90 ± 0.01^c^
Total sugars^d^	50.10 ± 4.22^a^	29.98 ± 2.75^b^	23.89 ± 0.56^c^

^a,b,c^In each row, the different letters represent significant differences between samples (p < 0.05); ^d^ % or g/100 g dw; ^e^ kcal/100 g

The analytical results on free sugars (Table 1) indicated that sorbitol, trehalose, glucose and inositol could be detected in our three truffle samples, and sorbitol and trehalose were the main free sugars; the contents of total sugar ranged from 23.89 to 50.10 g/100 g dw. The main free sugars found in *T. latisporum* were sorbitol (33.82 g/100 g dw) and trehalose (13.84 g/100 g dw), followed by glucose (1.25 g/100 g dw) and inositol (1.18 g/100 g dw); in *T. subglobosum* dominant were sorbitol (22.65 g/100 g dw) and trehalose (4.41 g/100 g dw), followed by inositol (2.00 g/100 g dw) and glucose (0.92 g/100 g dw). In *T. pseudohimalayense*,the main free sugars were sorbitol (17.55 g/100 g dw) and trehalose (4.22 g/100 g dw), followed by glucose (1.22 g/100 g dw) and inositol (0.90 g/100 g dw). *T. latisporum* showed the highest concentration of trehalose, sorbitol and total sugar; *T. subglobosum* presented the highest level of inositol; while *T. pseudohimalayense* was observed with a low trehalose, sorbitol, inositol and total sugar concentration.

Table 2 showed the distribution of saturated fatty acids (SFA), monounsaturated fatty acids (MUFA), and polyunsaturated fatty acids (PUFA) in the studied lyophilized truffles samples. The results showed that PUFA (from 48.28 to 59.91%) were the main group of FA, followed by MUFA (from 23.17 to 35.50%) and then SFA (from 16.22 to 16.91%). The linoleic acid is a main component of PUFA (linoleic acid levels: from 46.33 to 51.49% of total FA; PUFA level: from 48.28 to 59.91% of total FA). Linoleic acid (C18:2n6c), oleic acids (C18:1n9), stearic acids (C18:0) and palmitic acids (C16:0) were the most abundant in the three *Tuber* truffles. *Tuber latisporum s*howed the highest contents of total FA and UFA, while *T. subglobosum* had the lowest contents; and in *T. pseudohimalayense* the contents were close to, but slightly lower than, that of *T. latisporum*. The content of PUFA (367.98 mg/100 g dw) in *T. pseudohimalayense* was very close to *T. latisporum* (360.61 mg/100 g dw), the lowest was present in *T. subglobosum*.

**Table 2:** Distribution of individual fatty acids (mg) in dried powder (100 g) formulations of three *Tuber* species

Fatty acids	*T. latisporum*	*T. subglobosum*	*T. pseudohimalayense*
C14:0	nd	0.59 ± 0.03^a^	nd
C15:0	nd	0.44 ± 0.01^a^	nd
C16:0	72.36 ± 0.41^b^	41.96 ± 0.36^c^	80.93 ± 1.30^a^
C16:1	1.06 ± 0.00^c^	1.63 ± 0.01^b^	3.32 ± 0.09^a^
C17:0	nd	0.58 ± 0.00^b^	0.72 ± 0.02^a^
C18:0	47.56 ± 1.75^a^	26.06 ± 0.51^c^	33.18 ± 0.48^b^
C18:1n9c	250.97 ± 0.99^a^	94.04 ± 0.96^c^	219.63 ± 2.02^b^
C18:2n6c	347.47 ± 0.07^b^	246.81 ± 0.00^c^	365.24 ± 0.16^a^
C18:3n3	9.52 ± 0.13^a^	nd	nd
C20:0	1.01 ± 0.02^a^	0.75 ± 0.01^c^	0.86 ± 0.01^b^
C20:1c	13.15 ± 0.30^a^	0.78 ± 0.01^b^	1.00 ± 0.01^b^
C20:2c	2.99 ± 0.11^a^	1.41 ± 0.02^b^	1.41 ± 0.03^b^
C20:4n6	0.64 ± 0.01^b^	0.63 ± 0.02^b^	0.81 ± 0.02^a^
C20:5n3	nd	0.54 ± 0.02^a^	0.53 ± 0.01^a^
C22:0	nd	nd	0.97 ± 0.01^a^
C24:0	nd	nd	0.78 ± 0.00^a^
SFA^1^	121.17 ± 2.16^a^	70.38 ± 0.58^c^	117.35 ± 1.35^b^
MUFA^f^	265.19 ± 0.70^a^	96.46 ± 0.96^c^	223.95 ± 1.92^b^
PUFA^f^	360.61 ± 0.08^b^	249.39 ± 0.03^c^	367.98 ± 0.19^a^
SFA^g^	16.22 ± 0.23^c^	16.91 ± 0.11^a^	16.56 ± 0.18^b^
MUFA^g^	35.50 ± 0.06^a^	23.17 ± 0.17^c^	31.57 ± 0.23^b^
PUFA^g^	48.28 ± 0.18^c^	59.91 ± 0.18^a^	51.87 ± 0.11^b^

Myristic acid (C14:0); pentadecanoic acid (C15:0); palmitic acid (C16:0); palmitoleic acid (C16:1); heptadecanoic acid (C17:0); stearic acid (C18:0); oleic acid (C18:1n9c); linoleic acid (C18:2n6c); *α*-linolenic acid (C18:3n3); arachidic acid (C20:0); cis-11-eicosenoic acid (C20:1c); cis-11,14-eicosadienoic acid (C20:2c); arachidonic acid (C20:4n6); eicosapentaenoic acid (C20:5n3); behenic acid (C22:0); lignoceric acid (C24:0)

*nd* not detected

^a,b,c^In each row, the different letters represent significant differences between samples (p < 0.05); ^d^ % or g/100 g dw; ^e^ kcal/100 g; ^f^ mg/100 g dw; ^g^ % of total FA

### Bioactive compounds and antioxidant activity of methanol extracts

Table 3 presents phenols, flavonoids, ascorbic acid, β-carotenoid, lycopene and phenolic acids concentrations contained in methanol extract of the three *Tuber* samples. Phenols, flavonoids, β-carotenoid and lycopene were the major bioactive components in these samples. Extraction of *T. pseudohimalayense* showed a highest content of phenols (735.01 mg of GAE/100 g extract), whereas that of *T. subglobosum* and *T. latisporum* were lower and close to each other (450.2 mg of GAE/100 g extract) and 446.6 mg of GAE/100 g extract, respectively). Extraction of *T. subglobosum* showed a highest content of flavonoids (1.355 g of rutin/100 g extract), followed by *T. latisporum* (900.2 mg of rutin/100 g extract) and then *T. pseudohimalayense* (611.3 mg of rutin/100 g extract). Extraction of *T. pseudohimalayense* revealed a high content in β-carotenoid and lycopene (271.3 mg of β-carotene/100 g extract and 355.2 mg/100 g extract, respectively), followed by *T. latisporum* (170.6 mg of β-carotene/100 g extract and 236.0 mg/100 g extract, respectively) and then *T. subglobosum* (144.3 mg of β-carotene/100 g extract and 151.62 mg/100 g extract, respectively). All three *Tuber* samples showed a similar content of ascorbic acid, within the range of 4.59–4.63 g/100 g extract. Moreover, four phenolic compounds, gallic acid, 4-hydroxybenzoic acid, 3,4-dihydroxybenzaldehyde and homogentisic acid were identified in these truffles (Table 3). Of them, homogentisic acid was detected in all samples, with a high concentration in *T. latisporum* (61.03 mg/100 g extract) and *T. pseudohimalayense* (64.13 mg/100 g extract) and a moderate level in *T. subglobosum* (23.03 mg/100 g extract); 3,4-dihydroxybenzaldehyde was just found in *T. latisporum* (62.71 mg/100 g extract); gallic acid was detected in *T. subglobosum* (40.87 mg/100 g extract) and *T. pseudohimalayense* (39.83 mg/100 g extract); 4-hydroxybenzoic acid was found in *T. latisporum* (24.84 mg/100 g extract) and *T. pseudohimalayense* (21.93 mg/100 g extract); gallic acid and 4-hydroxybenzoic acid were all not detected (<LOD) in *T. latisporum* and *T. subglobosum*.

**Table 3:** Content of antioxidant compounds in the methanol extract of freeze-dried powder of three *Tuber* species

	*T. latisporum*	*T. subglobosum*	*T. pseudohimalayense*
Total phenols^h^	446.60 ± 3.8^b^	450.20 ± 3.4^b^	735.01 ± 2.5^a^
Total flavonoids^i^	900.22 ± 14.72^b^	1355.43 ± 18.01^a^	611.26 ± 7.40^c^
Vitamin C^j^	4.63 ± 0.01^a^	4.61 ± 0.01^ab^	4.59 ± 0.02^b^
Total carotenoids^k^	170.63 ± 3.06^b^	144.33 ± 0.87^c^	271.33 ± 0.66^a^
Lycopene^j^	236.03 ± 0.80^b^	151.62 ± 0.32^c^	355.18 ± 0.46^a^
Gallic^j^	nd	40.87 ± 0.42^b^	39.83 ± 0.46^a^
4-Hydroxybenzoic^j^	24.84 ± 0.05^a^	nd	21.93 ± 0.63^b^
3,4-Dihydroxybenzaldehyde^j^	62.71 ± 0.11^a^	nd	nd
Homogentisic acid^j^	61.03 ± 0.35^a^	23.03 ± 015^c^	64.13 ± 0.25^b^
*p*-Coumaric acid^j^	nd	nd	nd
*o*-Coumaric acid^j^	nd	nd	nd

*nd* not detected

^a,b,c^In each row, the different letters represent significant differences between samples (p < 0.05); ^d^ % or g/100 g dw; ^e^ kcal/100 g; ^f^ mg/100 g dw; ^g^ % of total FA; ^h^ mg of GAE/100 g extract; ^i^ mg of Rutin/100 g extract; ^j^ g/100 g extract; ^k^ mg of β-carotene/100 g extract

The antioxidant activity of the extracts obtained from our truffle powder formulations was evaluated by different in vitro assays (Table 4). *Tuber latisporum* showed the most efficient reducing power measured by ferrous ion-chelating assay and ferricyanide/prussian blue assay, which is compatible to the variety and content of phenolic acids. Ferrous ion-chelating activity of *T. subglobosum* was higher than that of *T. pseudohimalayense*. Ferricyanide/Prussian blue assay of our three truffles showed a general reducing power (EC_50_: 12.95–20.02 mg/mL). *Tuber pseudohimalayense* was the most efficient species concerning radical scavenging activity measured by DPPH scavenging activity and hydroxyl radical-scavenging assay. *T. latisporum* presented lower radical scavenging property than *T. pseudohimalayense*, which is compatible to its lower total phenols, β-carotenoid and lycopene content (Table 3).

**Table 4:** Antioxidant activity of the methanol extracts obtained from dried powder formulations of three *Tuber* species from China

Antioxidant properties	Assay	EC_50_ value (mg/mL)		
		*T. latisporum*	*T. subglobosum*	*T. pseudohimalayense*
Reducing power	Ferrous ion-chelating	0.82 ± 0.07^c^	1.50 ± 0.08^b^	1.92 ± 0.13^a^
	Ferricyanide/Prussian blue	12.95 ± 0.03^c^	13.69 ± 0.12^b^	20.02 ± 0.10^a^
Radical scavenging	DPPH	6.94 ± 0.15^b^	7.87 ± 0.15^a^	6.03 ± 0.26^c^
	Hydroxyl radical	1.29 ± 0.03^a^	1.32 ± 0.04^a^	1.11 ± 0.04^b^

The results are presented in EC_50_ values, meaning that higher values correspond to lower reducing power, radical scavenging activity. EC_50_ is the concentration of the extract that corresponds to 50% of antioxidant activity for the ferrous ion-chelating, DPPH, hydroxy, and or 0.5 of absorbance for the ferricyanide/Prussian blue assays

^a,b,c^In each row, the different letters represent significant differences between samples (p < 0.05)

## Discussion

Although many edible mushrooms, including truffles, are the subject of scientific studies that confirm their benefits, there are many species that are still poorly explored. In this study, the powder formulations of the three *Tuber* species revealed the presence of essential nutrients such as proteins, carbohydrates and UFA. They present low fat content and high content of protein as well as unsaturated fatty acid, which is beneficial to human health.

Of our three truffles, the white truffle *T. latisporum* showed the highest content of protein (14.64%), followed by the red truffle *T. pseudohimalayense* (14.28%), they are higher than the value reported for black truffles *T. aestivum* (11–12.9%) and *T. melanosporum* (7.5–8.7%) by Saltarelli et al. (2008); the protein of pale yellow truffle *T. subglobosum* (10.96%) was higher than that of black truffles *T. melanosporum* (7.5–8.7%). Saltarelli et al. (2008) evidenced white truffle *T. magnatum* from Italy are rich source of proteins (20.5–24%). With regard to desert truffle, Sawaya et al. (1985) compared the compositional difference amongst three Saudi Arabian truffles including two black desert truffles Gibaah and Kholeissi (both are *Terfezia claveryi* according to Hussain and Al-Ruqaie 1999) and one white truffle *Tirmania nivea* (Zubaidi). They found these truffles’ protein contents ranged from 19.59 to 27.18%, and the white truffle *T. nivea* had the highest protein content. These remind us that white truffles probably contain more protein.

Beara et al. (2014) determinate gallic acid of 0.2 μg/g dw in the methanol extract of *T. magnatum* from Serbia, which was remarkably lower than that in *T. subglobosum* (9.11 μg/g dw) and *T. pseudohimalayense* (10.16 μg/g dw) in this study. Phenolic compounds in *T. melanosporum*, *T. aestivum* and *T. indicum* from Soria (Spain) were analysed by Villares et al. (2012). Gallic acid was found in *T. aestivum* (63.65 μg/g) and *T. indicum* (61.54 μg/g), which were significantly lower than in *T. subglobosum* (40.87 mg/100 g extract) and *T. pseudohimalayense* (39.83 mg/100 g extract).

Linoleic acid (C18:2n6c), oleic acids (C18:1n-9), stearic acids (C18:0) and palmitic acids (C16:0) were the most abundant in three *Tuber* truffles. Previous studies on FA of *Tuber* species, including *T. texense* (Beuchat et al. 1993), *T. melanosporum* (Harki et al. 2006), *T. magnatum* (Angelini et al. 2015), *T. aestivum* (Tang et al. 2011; Angelini et al. 2015), *T. indicum*, *T. himalayense*, and *T. borchii* (Tang et al. 2011), also showed that the major ingredients of FA were UFA, particularly include linoleic acid and oleic acid. Noteworthily, Tang et al. (2011) first discovered arachidonic, eicosapentaenoic (EPA), docosahexaenoic (DHA), and γ-linolenic acids in *Tuber* species, and two of them, arachidonic and EPA, were identified in this study. Furthermore, *α*-linolenic acid (ALA), as the precursor of EPA and DHA, also appeared in *T. latisporum*.

The three truffles all showed high antioxidant activity, especially *T. latisporum* and *T. pseudohimalayense*, which were also richer in phenolic compounds than *T. subglobosum*. Compared with our three truffles, *T. indicum* (EC_50_ > 30 mg/mL) with a lower levels of total phenols (2.62 mg GAE/g) and flavonoids (1.97 mg Rutin/g extract) had a lower reducing power and a lower hydroxyl radical scavenging activity (EC_50_: 25.6 mg/mL) (Guo et al. 2011). However, *T. indicum* could be good at scavenging DPPH radical (EC_50_: 1.61 mg/mL), whereas our three truffles more tend to scavenge hydroxyl radical (EC_50_: 1.11–1.32 mg/mL) (Guo et al. 2011).

There is a relationship between antioxidant activity and the concentrations of total phenol, total flavonoid and phenolic acids, but not a linear relationship. The antioxidant action may be raised by other substances such as tocopherols and β-carotene (Cheung et al. 2003). We need more effort to know what kind of material is played a key role in the reaction. All in all, the studied truffle power formulations might be useful as nutraceuticals and antioxidant-rich supplements.

## Abbreviations

GC–MS: gas chromatography–mass spectrometer
HPLC: high-performance liquid chromatography
ME: methanol extract
DAD: diode array detector
PUFA: polyunsaturated fatty acids
MUFA: monounsaturated fatty acids
SFA: saturated fatty acids
FA: fatty acids
UFA: unsaturated fatty acids

## References

[CR1] Angelini P, Tirillini B, Properzi A, Rol C, Venanzoni R (2015). Identification and bioactivity of the growth inhibitors in spp. methanolic extracts. Plant Biosyst.

[CR2] Barros L, Ferreira MJ, Queirós B, Ferreira IC, Baptista P (2007). Total phenols, ascorbic acid, β-carotene and lycopene in Portuguese wild edible mushrooms and their antioxidant activities. Food Chem.

[CR3] Beara IN, Lesjak MM, Četojević-Simin DD, Marjanović ZS, Ristić JD, Mrkonjić ZO, Mimica-Dukić NM (2014). Phenolic profile, antioxidant, anti-inflammatory and cytotoxic activities of black (*Tuber aestivum* Vittad.) and white (*Tuber magnatum* Pico) truffles. Food Chem.

[CR4] Beuchat LR, Brenneman TB, Dove CR (1993). Composition of the pecan truffle (*Tuber texense*). Food Chem.

[CR5] Bokhary HA, Sarwat P (1993). Chemical composition of desert truffles *Terfezia claveryi*. J Food Compos Anal.

[CR6] Carneiro AA, Ferreira IC, Dueñas M, Barros L, da Silva R, Gomes E, Santos-Buelga C (2013). Chemical composition and antioxidant activity of dried powder formulations of *Agaricus blazei* and *Lentinus edodes*. Food Chem.

[CR7] Cheung LM, Cheung PCK, Ooi VCE (2003). Antioxidant activity and total phenolics of edible mushroom extracts. Food Chem.

[CR8] Culleré L, Ferreira V, Chevret B, Venturini E, Sánchez-Gimeno AC, Blanco D (2010). Characterisation of aroma active compounds in black truffles (*Tuber melanosporum*) and summer truffles (*Tuber aestivum*) by gas chromatography–olfactometry. Food Chem.

[CR9] Dubost NJ, Ou B, Beelman RB (2007). Quantification of polyphenols and ergothioneine in cultivated mushroom and correlation to total antioxidant capacity. Food Chem.

[CR10] Dundar A, Yesil OF, Acay H, Okumus V, Ozdemir S, Yildiz A (2012). Antioxidant properties, chemical composition and nutritional value of *Terfezia boudieri* (Chatin) from Turkey. Food Sci Technol Int.

[CR11] Gao JM, Liu HL, Liu JK (2001). A novel sterol from Chinese truffles *Tuber indicum*. Steroids.

[CR12] Gao JM, Wang CY, Zhang AL, Liu JK (2001). A new trihydroxy fatty acid from the ascomycete. Chinese truffle *Tuber indicum*. Lipids.

[CR13] Guo T, Wei L, Sun J, Hou CL, Fan L (2011). Antioxidant activities of extract and fractions from *Tuber indicum* Cooke & Massee. Food Chem.

[CR14] Hall IR, Yun W, Amicucci A (2003). Cultivation of edible ectomycorrhizal mushrooms. Trends Biotechnol.

[CR15] Hamza A, Zouari N, Zouari S, Jdir H, Zaidi S, Gtari M, Neffti M (2016). Nutraceutical potential, antioxidant and antibacterial activities of *Terfezia boudieri* Chatin, a wild edible desert truffle from Tunisia arid zone. Arabian J Chem.

[CR16] Harki E, Bouya D, Dargent R (2006). Maturation-associated alterations of the biochemical characteristics of the black truffle *Tuber melanosporum* Vitt. Food Chem.

[CR17] Hussain G, Alruqaie IM (1999). Occurrence, chemical composition, and nutritional value of truffles: an overview. PJBS.

[CR19] Kehrer JP (1993). Free radicals as mediators of tissue injury and disease. Crit Rev Toxicol.

[CR20] Klein BP, Perry AK (1982). Ascorbic acid and vitamin A activity in selected vegetables from different geographical areas of the United States. J Food Sci.

[CR21] Lee J, Koo N, Min DB (2004). Reactive oxygen species, aging, and antioxidative nutraceuticals. Compr Rev Food Sci Food Saf.

[CR22] Murcia MA, Martinez-Tome M, Jimenez A, Vera A, Honrubia M, Parras P (2002). Antioxidant activity of edible fungi (truffles and mushrooms): losses during industrial processing. J Food Prot.

[CR23] Saltarelli R, Ceccaroli P, Cesari P, Barbieri E, Stocchi V (2008). Effect of storage on biochemical and microbiological parameters of edible truffle species. Food Chem.

[CR24] Sawaya WN, Al-Shalhat A, Al-Soair A, Al-Mahammad M (1985). Chemical composition and nutritional values of truffles of Saudi Arabia. J Food Sci.

[CR25] Stojković D, Reis FS, Ferreira IC, Barros L, Glamočlija J, Ćirić A, Nikolić M, Stević T, Giveli A, Soković M (2013). *Tirmania pinoyi*: chemical composition, in vitro antioxidant and antibacterial activities and in situ control of *Staphylococcus aureus* in chicken soup. Food Res Int.

[CR26] Tang Y, Li YY, Li HM, Wan DJ, Tang YJ (2011). Comparison of lipid content and fatty acid composition between tuber fermentation mycelia and natural fruiting bodies. J Agric Food Chem.

[CR27] Tang Y, Li HM, Tang YJ (2012). Comparison of sterol composition between *Tuber fermentation* mycelia and natural fruiting bodies. Food Chem.

[CR28] Villares A, García-Lafuente A, Guillamón E, Ramos À (2012). Identification and quantification of ergosterol and phenolic compounds occurring in *Tuber* spp. truffles. J Food Compos Anal.

[CR29] Wang S, Marcone MF (2011). The biochemistry and biological properties of the world’s most expensive underground edible mushroom: truffles. Food Res Int.

